# Getting Lost Behavior in Patients with Mild Alzheimer’s Disease: A Cognitive and Anatomical Model

**DOI:** 10.3389/fmed.2017.00201

**Published:** 2017-11-16

**Authors:** Chathuri Yatawara, Daryl Renick Lee, Levinia Lim, Juan Zhou, Nagaendran Kandiah

**Affiliations:** ^1^Department of Neurology, National Neuroscience Institute, Singapore, Singapore; ^2^Duke—NUS Medical School, Singapore, Singapore

**Keywords:** Alzheimer’s disease, getting lost, wayfinding, medial temporal lobe, top-down modulation

## Abstract

**Background:**

Getting lost behavior (GLB) in the elderly is believed to involve poor top-down modulation of visuospatial processing, by impaired executive functions. However, since healthy elderly and elderly with Alzheimer’s disease (AD) experience a different pattern of cognitive decline, it remains unclear whether this hypothesis can explain GLB in dementia.

**Objective:**

We sought to identify whether poor executive functions and working memory modulate the relationship between visuospatial processing and prevalence of GLB in healthy elderly and patients with AD. Complementary to this, we explored whether brain regions critical for executive functions modulate the relationship between GLB and brain regions critical for visuospatial processing.

**Method:**

Ninety-two participants with mild AD and 46 healthy age-matched controls underwent neuropsychological assessment and a structural MRI. GLB was assessed using a semistructured clinical interview. Path analysis was used to explore interactions between visuospatial deficits, executive dysfunction/working memory, and prevalence of GLB, in AD and controls independently.

**Results:**

For both healthy controls and patients with mild AD, visuospatial processing deficits were associated with GLB only in the presence of poor working memory. Anatomically, GLB was associated with medial temporal atrophy in patients with mild AD, which was not strengthened by low frontal gray matter (GM) volume as predicted. Instead, medial temporal atrophy was more strongly related to GLB in patients with high frontal GM volumes. For controls, GLB was not associated with occipital, parietal, medial temporal, or frontal GM volume.

**Conclusion:**

Cognitively, a top-down modulation deficit may drive GLB in both healthy elderly and patients with mild AD. This modulation effect may be localized in the medial temporal lobe for patients with mild AD. Thus, anatomical substrates of GLB in mild AD may not follow the typical top-down modulation mechanisms often reported in the healthy aging population. Implications advance therapeutic practices by highlighting the need to target both working memory and visuospatial deficits simultaneously, and that anatomical substrates of GLB may be disease specific.

## Introduction

Getting lost behavior (GLB) is defined as the inability to find one’s way in familiar or unfamiliar environments (1). GLB is highly prevalent in patients with Alzheimer’s disease (AD), with an approximate 40% of patients reportedly experiencing some phenomenon of getting lost (2). This prevalence increases to 70% in patients with severe AD and often leads to institutionalization, increased risk of falls and even death (3). Despite its prevalence and devastating impact, the mechanisms underlying GLB in AD remain unclear.

Early assumptions on the underlying cause of GLB in patients with AD have focused on visuospatial processing problems, such as motion perception that guides self-movement and maintains spatial orientation (4). However, more recent speculations have centered around GLB as a problem with higher level cognition such as working memory, defined as the capacity to temporarily maintain and manipulate information in memory, and executive functions, which involve mental flexibility, problem solving and decision making (5).

One theory that integrates the functions of both visuospatial processing and higher level cognition is the top-down modulation hypothesis of cognitive aging. This hypothesis proposes that working memory and executive functions may exert modulatory control over the efficacy of visuospatial processing and its association with behavioral systems such as navigation (6). For example, if lost, selective attention is required to moderate visual perception toward relevant visual information and suppress attention toward irrelevant information competing for cognitive resources, additionally, mental flexibility is required to facilitate strategy switching and working memory is required to engage visual memory to manipulate information no longer in the environment (5).

The neural basis for higher level cognition and visuospatial processing are anatomically distinct, with the former localized in the frontal brain region (7, 8) and the latter localized in the occipital, posterior parietal and medial temporal brain regions (9). Despite the distinct locations, the regions for higher level cognition and visuospatial processing are functionally integrated (10). For instance, a fMRI study in healthy adults demonstrated that the frontal cortex modulated the magnitude of activity in the occipital cortex during a delayed visual recognition task (11). Following on from this, a subsequent study identified that the magnitude of this modulation predicted successful performance on a visuospatial processing task (12).

In healthy cognitive aging, frontal lobe structures are typically the first to deteriorate (13). As a result, the elderly demonstrate a pronounced deficit in suppressing cortical activity associated with task-irrelevant information processing, compared to younger adults (6). This deficit with top-down modulation of cortical activity is believed to be one substrate of GLB in the elderly (14). Compared to healthy cognitive aging, patients with AD experience early atrophy in structures critical for learning and memory, such as the parietal and medial temporal lobe, while structures important for top-down modulation, such as the frontal lobe, may become affected at later stages of the disease (15). Due to the different patterns of neurodegeneration associated with AD compared to healthy aging, it remains unclear whether the top-down hypothesis is suitable for explaining GLB in patients with mild AD.

We sought to identify whether deficits with higher order cognition, such as executive functions and working memory, moderate the effect of visuospatial deficits on prevalence of GLB. This hypothesis was explored in healthy controls and patients with AD to identify whether the same mechanisms are present in the normal and abnormal aging process. Complimentary to this, we sought to identify whether anatomical mechanisms of GLB in patients with mild AD involve top-down modulation deficits. Specifically, we predicted that reduced volume of frontal gray matter (GM) may strengthen the relationship between GLB and atrophy in regions critical for visuospatial processing, namely the occipital, parietal and medial temporal GM.

## Materials and Methods

### Sampling, Screening, Procedure

In this cross-sectional study, patients with mild AD were recruited from a tertiary neurology center in Singapore (National Neuroscience Institute) between 2013 and 2016. Diagnosis of mild probable AD was based on the NIA-AA Criteria (16) and a full medical work-up, which involved medical and caregiver reports, structural MRI, a comprehensive cognitive evaluation and blood test to rule out cognitive impairment due to vitamin deficiency or thyroid abnormalities. Additional criteria for a diagnosis of mild AD included a Clinical Dementia Rating Scale (CDR) (17) of 1. Age-matched controls from the community were recruited at the National Neuroscience Institute from 2010 to 2016 and included elderly who were “cognitively normal,” as determined by a comprehensive cognitive assessment, a MMSE score >28 and a CDR of 0. Recruitment of the clinical and control cohort was non-random and involved consecutive sampling methods.

Exclusion criteria for all participants included (a) major visual impairment, such as blindness, visual agnosia or cortical blindness, (b) a current diagnosis or history of neuropsychiatric conditions (e.g., psychosis, depression), (c) comorbid neurodegenerative diseases (e.g., Parkinson’s disease), (d) a history of clinical strokes (e.g., CAA and prior clinical strokes), and (e) a current or history of alcohol or drug abuse.

### Measures

*Primary outcome measure*, GLB, was indexed using a semistructured clinical interview with a psychologist blinded to diagnosis. The interview queried the changes, if any, in the visuospatial abilities of the subject and involved responses from both the subject and their caregiver, or family member in the case of controls, for clarification purposes. The subject was queried on whether they still travel alone, how well they can recall travel routes (including travel route to the present location), whether they make wrong turns on familiar paths and whether they have experienced getting lost in the past 6 months. Caregiver/family member questions sought to validate the subject’s responses and focused on whether the subject still travels on their own and whether there has been instances of making wrong turns on familiar routes or getting lost in the past 6 months (see supplementary materials for full interview). After cross-referencing the accounts of the subject and caregiver/family member, the presence of GLB was then recorded as a yes or no by the psychologist based on clear indications that the subject was not able to orientate themselves in familiar environments, or that there have been instances of getting lost.

*Cognitive predictor variable*s included working memory, which was indexed using the composite score of Wechsler’s forward and backward digit span tasks [WMS-IV; (18)]; executive function, which was indexed using the composite score of the Frontal Assessment Battery [FAB; (19)] and Color Trails 2 task (20); and visuospatial processing, which was indexed using the composite of Wechsler’s block design [WAIS–IV; (21)] and Wechsler’s immediate and delayed visual reproduction task [Wechsler Memory Scale-IV; (18)].

### Image Acquisition and Processing

*Anatomical predictor variables* included the volumetric measure of frontal, parietal, medial temporal and occipital GM. Subjects underwent MRI in a whole body MR system which included a 3T Siemens Tim Trio system (Siemens, Erlangen, Germany) and a 3T Siemens Prisma system (Siemens, Erlangen, Germany). Voxel-based morphometry was conducted using the Computational Anatomy Toolbox (CAT12) package for the Statistical Parametric Mapping 12 (SPM12) software (http://www.fil.ion.ucl.ac.uk/spm) in MATLAB. Volumetric MPRAGE sequences were converted from DICOM to 3D NIFTI format and manually oriented to be within the standard Montreal Neurological Institute template space. Images were segmented into GM and cerebrospinal fluid maps using a unified segmentation pipeline (22), including affine regularization to the International Consortium for Brain Mapping space template for East Asian brains, bias corrections, and affine and non-linear modulated normalization. The generated GM maps were then smoothed (8 mm full width at half maximum) in SPM12. CAT12 was used to estimate the total intracranial volume for each subject, and the smoothed GM maps were used to generate global volumes of GM, and also regional volumes based on regions of interest defined using the Wake Forest University Pick Atlas v3.0 software toolbox (23).

### Statistical Analysis

#### Group Comparisons

A *t*-test was used to identify the neuropsychological and anatomical deficits in the mild AD group as compared to age-matched healthy controls.

#### Path Analysis

**Figure 1:**
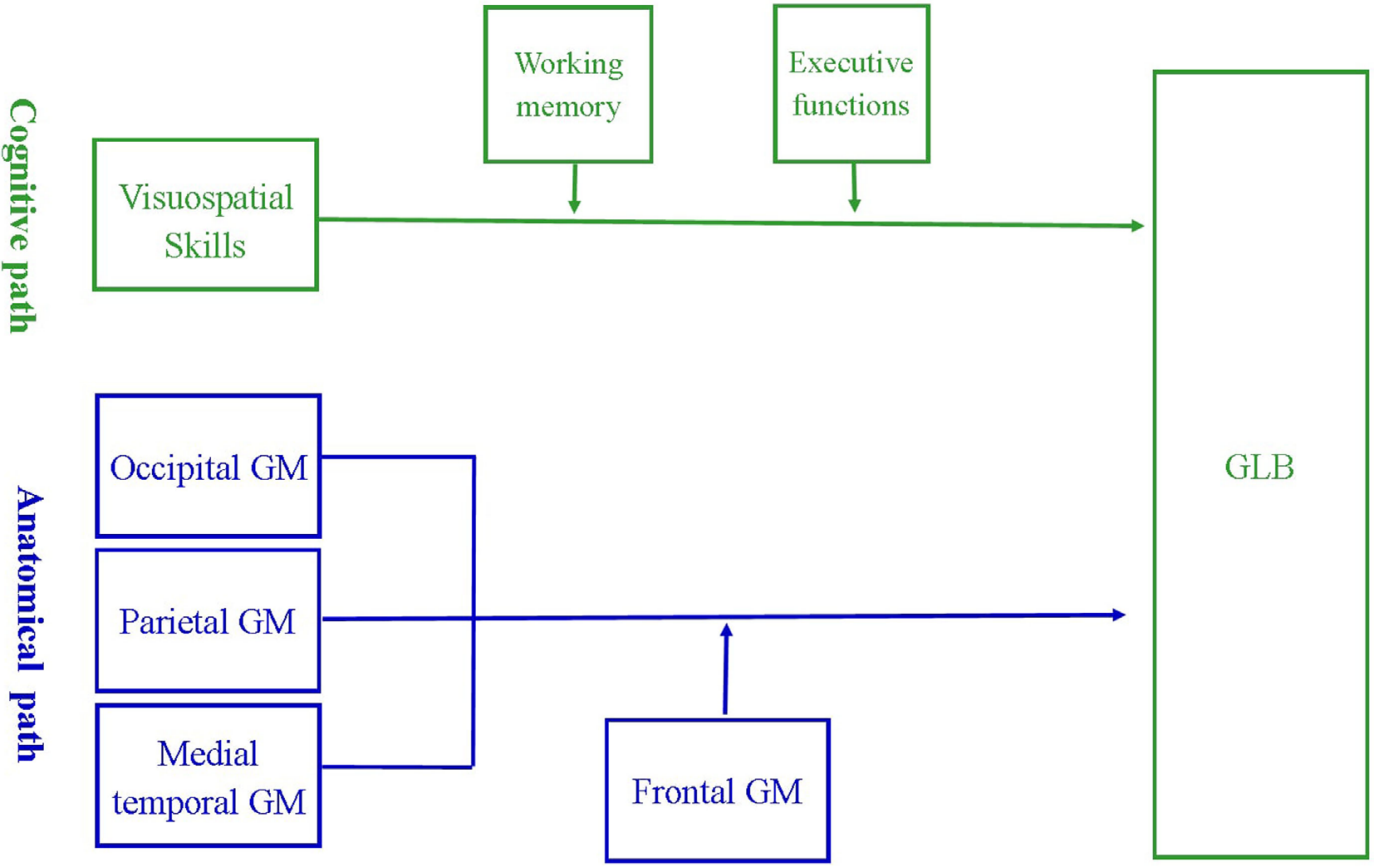
*A priori* model of the cognitive and anatomical pathways associated with getting lost behavior (GLB). The cognitive model suggests that the relationship between visuospatial deficits and prevalence of GLB may be strengthened by poor working memory and executive dysfunctions. The anatomical model suggests that reduced volume of frontal gray matter (GM) may strengthen the relationship between GLB and atrophy in regions critical for visuospatial processing, namely occipital, parietal and medial temporal GM.

The *a priori* cognitive and anatomical models, depicted in Figure 1, were assessed using moderation path analysis with SPSS Amos version 20 (24). Moderation analysis determined whether the effect of a predictor variable on an outcome was enhanced or attenuated in the presence of a third moderating variable. In our cognitive model, the predictor variables included visuospatial skills, the outcome included prevalence of GLB and the moderating variables included executive functions or working memory. In our anatomical model, the predictor variables included regions of visuospatial processing, namely the parietal, occipital or medial temporal GM, the outcome included prevalence of GLB and the moderating variable included the region for higher order cognition, namely frontal GM. The moderation effect was calculated by mean centering all variables, then multiplying each predictor variable with each moderating variable to obtain an “interaction variable.”

Path analysis was conducted separately for both the cognitive and anatomical models and for each diagnostic group (mild AD or controls) by including diagnosis type as the multi-group variable. For each analysis, the primary independent variable was the interaction variable, the secondary independent variables were the target predictor and moderating variable, while the dependent variable was the presence of GLB (indexed as a binary variable). Each analysis also controlled for years of education (given it was different between mild AD and controls), gender and MMSE score (given they were different between mild AD patients with and without GLB). The path analysis model fit was revised using modification indices and assessed using previously published recommended criteria: (a) χ^2^ *p* value > 0.05, (b) Bentler comparative fit index (CFI: >0.95), and (c) root mean error of approximation (RMSEA: <0.04) (25).

Due to our non-random sampling methods, we applied bias-corrected (BC) bootstrap estimation with 1,000 resamples as a non-parametric approach for estimating effect-sizes, SEs and biases (26). Bootstrapping is useful in regression because it measures the variability of the linear approximation of each path in the model and estimates the bias of this linear approximation to the population (27). BC bootstrap estimation has further been shown to be useful as a multiple comparison correction method for hypothesis testing (28–30). The significance of the BC bootstrap estimate was indicated by confidence intervals that did not contain 0. Effect sizes for the direct paths between independent and dependent variables were indexed using the standardized coefficient of the path, where 0.10 indicated a small effect, 0.30 indicated a medium effect and 0.50 indicated a large effect (31). Effect sizes for the path between the interaction and the dependent variable was indexed by squaring Cohen’s (31) estimations because interaction effects represent a product of two effects (32). Thus, a small interaction effect size would be 0.01, moderate would be 0.09, and large would be 0.25.

## Results

### Participants

The cohort consisted of 92 participants with mild AD and 46 healthy controls matched on age. Table 1 shows that compared to the controls, the mild AD group overall had less years of education (*p* = 0.00), a higher prevalence of GLB (*p* = 0.01), lower global cognition (*p* = 0.00), performed worse on all cognitive domain tasks (*p* = 0.00) and had significant atrophy in the medial temporal (*p* = 0.02) and occipital GM region (*p* = 0.00). Within the mild AD group, those that experienced GLB were more likely to be male (*p* = 0.01), have lower global cognition (*p* = 0.00), poorer performance on executive function tasks (FAB, *p* = 0.01 and color trials, *p* = 0.01), poorer performance on visuospatial tasks (block design, *p* = 0.001) and visual reproduction (immediate recall, *p* = 0.02) and reduced volumes in the medial temporal (*p* = 0.01) and occipital GM regions (*p* = 0.01). For healthy controls, no differences were observed between those with and without GLB.

**Table 1:** Participant characteristics.

	Mild AD, mean (SD)			Healthy controls, mean (SD)		
	Total (*N* = 92)	GLB+ (*N* = 26)	GLB− (*N* = 66)	Total (*N* = 46)	GLB+ (*N* = 4)	GLB− (*N* = 42)
**Demographics**						
Age (years)	68.30 (9.28)	71.14 (8.05)	67.18 (9.55)	65.32 (6.04)	63.67 (7.19)	65.48 (5.99)
Gender (males, %)	45 (48)	18 (69)	27 (41)	22 (48)	2 (50)	20 (48)
Years of education	9.62 (3.88)	9.62 (3.5)	9.62 (4.02)	13.02 (2.91)	11.50 (3.87)	13.17 (2.87)
Race						
Chinese	86	23	63	43	39	4
Malay	3	3	2	0	0	0
Indian	2	2	1	3	3	0
Other	1	1	0	0	0	0
GLB prevalence	26 (28%)	–	–	4 (8%)	–	–

**Cognitive measures**						
Global cognition						
MMSE (score range 0–30)	24.47 (4.39)	21.46 (4.82)	25.65 (3.61)	28.70 (1.57)	28.25 (0.97)	28.74 (1.64)
Executive function						
FAB (score range 0–18)	14.34 (3.11)	13.08 (2.56)	14.83 (3.18)	17.33 (0.96)	17.50 (5.77)	17.31 (1.00)
Color Trails 2 (seconds)	732.98 (135.65)	671.26 (185.28)	757.30 (102.15)	830.11 (26.91)	833.73 (7.01)	829.76 (28.13)
Working memory						
Digitspan-forward (score range 0–16)	9.25 (2.44)	8.62 (2.11)	9.50 (2.53)	11.04 (2.22)	10.50 (3.00)	11.10 (2.71)
Digitspan-backward (score range 0–16)	7.42 (2.09)	7.00 (1.60)	7.59 (2.16)	9.89 (3.08)	10.25 (3.30)	9.86 (3.09)
Visuospatial skills						
Block design (score range 0–48)	28.26 (11.23)	22.15 (10.68)	30.64 (10.58)	37.13 (7.05)	38.00 (6.92)	37.05 (7.14)
Immediate VR (score range 0–43)	25.15 (10.90)	20.77 (11.48)	26.88 (10.25)	36.07 (3.89)	36.75 (3.09)	36.00 (3.98)
Delayed VR (score range 0–43)	14.60 (12.77)	10.69 (11.84)	16.14 (12.88)	27.35 (9.44)	30.50 (4.43)	27.05 (9.76)

**Structural imaging (gray matter)**						
Frontal	71.77 (6.83)	69.84 (6.31)	72.52 (6.92)	73.64 (7.11)	77.55 (9.89)	73.26 (6.84)
Parietal	32.35 (3.41)	31.40 (3.4)	32.72 (3.46)	33.03 (3.16)	34.12 (5.57)	32.93 (2.92)
Medial temporal	46.11 (5.11)	43.76 (4.79)	40.80 (4.39)	48.22 (4.24)	49.15 (5.53)	48.14 (4.17)
Occipital	23.41 (2.93)	22.17 (2.69)	23.91 (2.97)	25.06 (2.86)	25.79 (4.34)	24.99 (2.85)

*AD, Alzheimer’s disease; GLB+, getting lost behavior was prevalent; GLB−, getting lost behavior was not prevalent; MMSE, mini-mental state examination; FAB, frontal assessment battery; VR, visual recall*.

### Path Analysis

#### Cognitive Model

The cognitive model had good model fit, with chi square (12) = 15.49, *p* = 0.21, CFI = 0.99 and RMSEA = 0.04. Table 2 presents the characteristics of each path in the model, after controlling for covariates. GLB was not directly associated with working memory, executive functions or visuospatial skills in patients with mild AD or healthy controls. The interaction between working memory and visuospatial skills was significantly associated with GLB for both groups, suggesting that visuospatial deficits were associated with GLB only for those with poor working memory. This interaction was of a moderate effect size for patients with mild AD and of a moderate to large effect size for healthy controls. The interaction between executive functions and visuospatial skills was not significant for either group.

**Table 2:** Regression coefficients and significance of paths in the cognitive model for patients with AD and healthy controls.

Relationships	Standardized *b*	SE	BC 95% CI
**Mild AD**			
Direct relationships			
Visuospatial skills → GLB	−0.17	0.14	−0.03 to 0.38
Working memory → GLB	−0.09	0.09	−0.22 to 0.04
Executive functions → GLB	−0.07	0.15	−0.33 to 0.17
Interactions			
Working memory × visuospatial skills → GLB	0.28	0.08	0.07–0.32*
Executive functions × visuospatial skills → GLB	−0.02	0.14	−0.28 to 0.82

**Healthy controls**			
Direct relationships			
Visuospatial skills → GLB	−0.22	0.26	−0.28 to 0.54
Working memory → GLB	−0.31	0.19	−0.67 to 0.01
Executive functions → GLB	−0.39	0.43	−0.10 to 1.4
Interactions			
Working memory × visuospatial skills → GLB	0.43	0.18	0.21–0.95**
Executive functions × visuospatial skills → GLB	−0.37	0.38	−0.83 to 0.43

*p < 0.05.

**p < 0.01.

*SE, standard error; BC, bias corrected; CI, confidence interval; GLB, getting lost behavior; AD, Alzheimer’s disease*.

#### Anatomical Model

**Figure 2:**
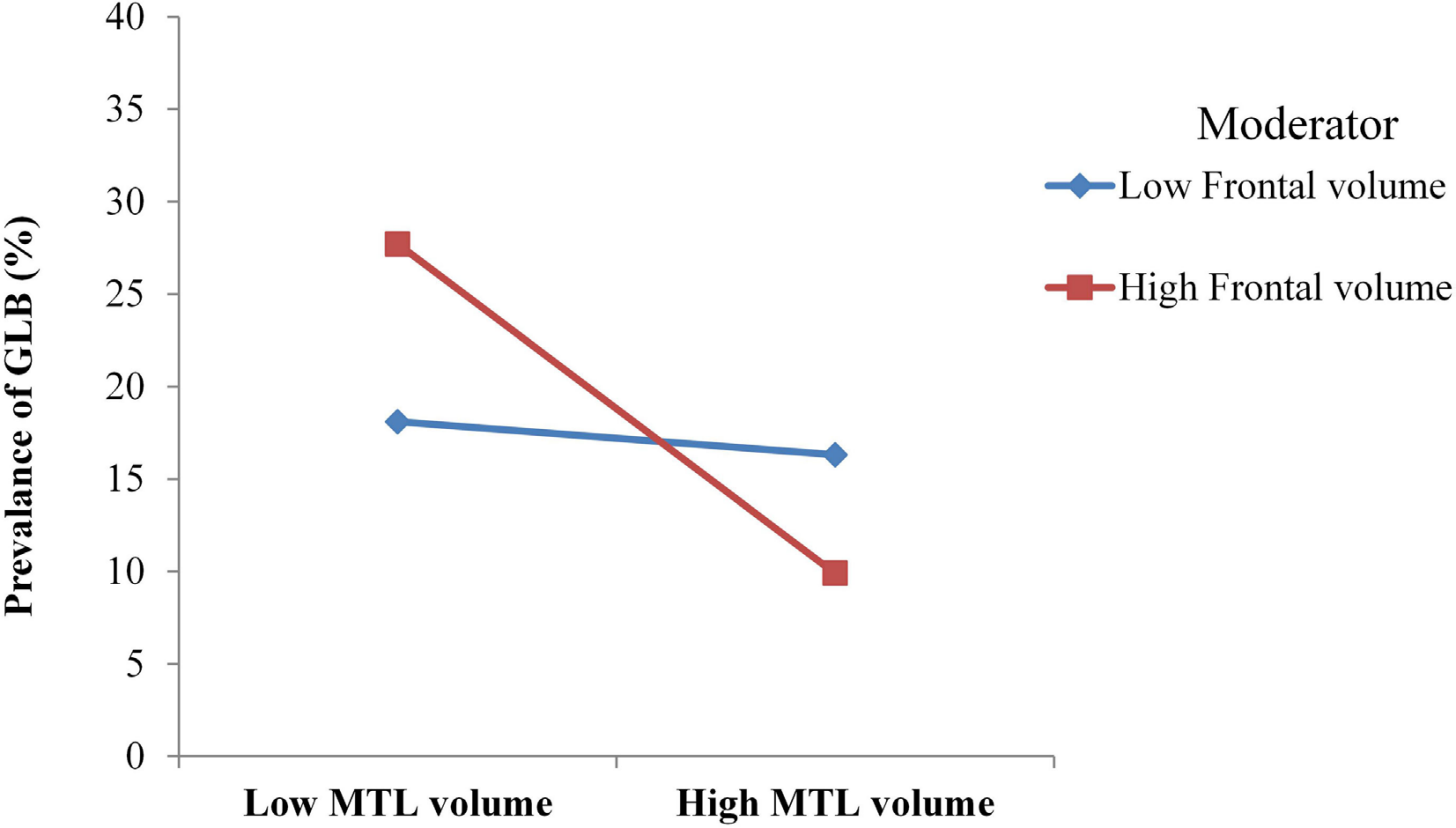
For mild Alzheimer’s disease patients with high volume of frontal gray matter (GM), medial temporal lobe (MTL) atrophy was more strongly associated with the prevalence of getting lost behavior (GLB). For patients with low frontal GM volume, no moderation was observed.

The anatomical model had good model fit, with χ^2^ (6) = 2.49, *p* = 0.47, CFI = 0.99 and RMSEA = 0.00. Table 3 depicts that for patients with mild AD, GLB was directly related to medial temporal GM, which was associated with a moderate to large effect size. GLB was not directly related to parietal, occipital or frontal GM (*p* > 0.05). The interaction between frontal and medial temporal GM was significant, see Figure 2. Frontal GM did not interact with parietal or occipital GM.

**Table 3:** Regression coefficients and significance of the paths in the anatomical model for patients with Alzheimer’s disease.

Relationships	Standardized *b*	SE	BC 95% CI
**Direct relationships**			
Occipital GM → GLB	−0.28	0.18	−0.61 to 0.02
Parietal GM → GLB	0.14	0.23	−0.24 to 0.52
Medial temporal GM → GLB	−0.45	0.21	−0.79 to −0.12^a^
Frontal GM → GLB	0.29	0.21	−0.17 to 0.55

**Interactions**			
Frontal GM × occipital GM → GLB	−0.27	0.12	−0.46 to 0.00
Frontal GM × parietal GM → GLB	−0.07	0.19	−0.41 to 0.23
Frontal GM × medial temporal GM → GLB	−0.22	0.17	−0.41 to −0.05*

*p < 0.05.

*SE, standard error; BC, bias corrected; CI, confidence interval; GLB, getting lost behavior; GM, gray matter*.

For healthy age-matched controls, GLB was not directly or indirectly related to frontal, parietal, medial temporal or occipital GM (*p* > 0.05).

## Discussion

### Main Findings

Getting lost behavior in patients with mild AD and healthy age-matched controls was associated with visuospatial processing deficits only in the presence of poor working memory, while controlling for educational attainment, gender and global cognition. This suggests that for both AD and normal aging, visuospatial processing deficits may not be sufficient for GLB, and that impairments with higher cognitive functions, including working memory, may be necessary. This finding is consistent with the hypothesis that GLB may involve a deficit with top-down modulation of visuospatial processing, by impaired working memory.

The anatomical substrates of GLB were not consistent with the top-down deficit hypothesis for neither patients with mild AD or healthy controls. In patients with mild AD, GLB was directly associated with medial temporal atrophy; however, this association was not strengthened in the presence of reduced frontal GM as predicted. Instead, the relationship between medial temporal atrophy and GLB was strengthened in patients with a high volume of frontal GM, indicating atypical modulatory mechanisms. Alternatively for healthy age-matched controls, GLB was not associated with occipital, parietal, medial temporal or frontal GM volumes, suggesting that the prevalence of GLB in this population may be associated with factors other than neural degeneration.

### Cognitive Model

Getting lost behavior in patients with mild AD was not directly associated with general cognitive functions, such as working memory, executive functions or visuospatial processing, which converges with previous findings (33). Some (33) have interpreted this lack of association to suggest that GLB may not be a manifestation of generalized cognitive decline, rather a navigation specific decline. An alternative perspective identified by our moderation analysis suggested that the interaction between cognitive functions may be critical for understanding GLB in mild AD rather than independent associations. More specifically, general visuospatial processing deficits may become associated with GLB in the context of poor working memory, whereby poor working memory may impede the encoding and manipulation of information necessary for visuospatial processing. These findings are consistent with behavioral studies demonstrating that GLB in mild AD is a primary function of poor spatial working memory, and that visuospatial information processing deficits are secondary to these deficits (34, 35). Thus, one cognitive mechanism of GLB in patients with mild AD may involve altered top-down modulation of visuospatial processing by poor working memory.

Similar to patients with AD, healthy age-matched controls exhibited an association between GLB and visuospatial processing deficits only in the context of poor working memory. Interestingly, insights from previous studies indicate that the groups may differ with the function of working memory in top-down modulation. For instance, in healthy aging, the inability to suppress task-irrelevant information is a key substrate of GLB (6). Meanwhile for patients with AD, the inability to store and manipulate task-relevant visuospatial information is believed to be primary for GLB (36). Thus, we propose that while the functional role of working memory in GLB may differ between healthy elderly and patients with mild AD, the mechanisms by which working memory deficits affect GLB are similar across the groups.

Contrary to expectations, executive functions did not play a top-down modulatory role on the relationship between visuospatial processing and GLB. Executive functions have been implicated in way-finding, which involves spatial problem solving abilities when appropriate solutions are not available in memory (5, 36). Our findings suggest that the information source used to problem solve, namely working memory, may be more critical for GLB in elderly with mild AD than the problem solving skill itself. Given that working memory deficits are a primary diagnostic feature of AD, we propose that cognitive functions most implicated in the top-down effects of GLB may be the most vulnerable cognitive functions in each disease group.

### Anatomical Model

Consistent with previous research (37), the medial temporal lobe was strongly associated with GLB in patients with mild AD. The effect size was moderate to large, suggesting that medial temporal atrophy may result in observable deficits with wayfinding in patients with mild AD. The medial temporal region includes the hippocampus, the subicular complex and the parahippocampal cortical regions, which collectively play a critical role in the encoding, storage and active manipulation of cognitive maps (38). Accordingly, past research has demonstrated that patients with lesions to the medial temporal lobe exhibit deficits with spatial memory, including recalling locations, drawing maps of the environment and making judgments about the distance and proximity of locations (39). Similar spatial memory deficits have been observed in patients with AD (33). Thus, together with previous literature, our findings suggest that structures controlling memory functions may be a primary anatomical substrate of GLB in patients with mild AD.

To advance our understanding of the anatomical mechanisms of GLB in patients with AD, we proposed that the association between medial temporal atrophy and GLB may be strengthened by the top-down effects of reduced frontal GM volume. Contrarily to this hypothesis, we observed that the association between medial temporal atrophy and GLB was strengthened in the presence of healthy frontal GM volume. In our cohort, patients with AD exhibited comparable volumes of frontal GM to the healthy controls, which is consistent with previous findings that the frontal lobe in AD often begins to degenerate at later stages of the disease (15). This may be one reason why patients with mild AD did not exhibit typical anatomical top-down modulation mechanisms as observed in healthy aging (6). Given that our cognitive model indicated that poor working memory was necessary for poor visuospatial deficits to be associated with GLB, it is likely that this cognitive top-down modulation in patients with mild AD may be localized in the medial temporal lobe.

The medial temporal lobe and posterior parietal lobe have been argued to have overlapping but complimentary roles in spatial navigation (33). However, we only observed the medial temporal lobe to be associated with GLB in patients with mild AD. One reason for this may be that only the medial temporal lobe was reduced in volume for mild AD patients compared to healthy age-matched controls, while the parietal GM appeared healthy. Given the medial temporal lobe is one of the first regions to become affected in AD (15), our findings suggest that disease-related patterns of atrophy may contribute to the vulnerability of the spatial navigation network in patients with mild AD. Thus, anatomical markers of GLB may be disparate for patients with mild AD and healthy elderly, stressing the need for tailored assessment criteria and treatment strategies.

### Strengths, Limitations, and Future Research

One strength of this study was that we used a real-world indicator of GLB, clinical interview. This measure was binary and future research may benefit from studying GLB as a continuous variable, which will allow the comparison of Alzheimer’s patients with GLB and without GLB. Such comparisons will identify neural correlates for GLB not contributed by anatomical changes accounted for by typical cognitive deficits such as episodic memory loss. Another strength is that we applied path analysis to assess simultaneous relationships between variables in a multivariable pathway, however we note that our cross-sectional design did not allow us to infer causality. Future research may benefit from investigating the predictive value of the cognitive and anatomical mechanisms on the incidence of GLB. One limitation of the current study was the non-random sampling procedure, which may limit the generalizability of the results. We further note that we explored broad neural regions while specific regions such as the dorsal occipital cortex, the posterior parietal cortex and the dorsolateral prefrontal cortex have previously been implicated in GLB (9, 40, 41). The inclusion of broad regions in the current study was an important preliminary step for model building, paving the way for future research to specify the models in more detail. We further note that the cognitive assessments were not navigation specific, however the trends were consistent with previous studies (33) utilizing navigation specific memory and visuospatial tasks.

### Conclusion

This study advanced our understanding of GLB by demonstrating that a cognitive top-down modulation deficit may drive GLB in both healthy elderly and patients with mild AD. Specifically, our findings suggest that visuospatial processing deficits may not be sufficient for GLB, and that its interaction with higher cognitive functions, including working memory, may be necessary. In patients with mild AD, GLB may be localized to disease-affected structures, such as the medial temporal lobe, and anatomical mechanisms of GLB may not involve typical top-down modulation. Implications of these cognitive and anatomical findings may advance the assessment and treatment of GLB in elderly with mild AD, including cognitive training, neurofeedback, neuromodulation, and pharmacological intervention. Specifically, intervention for GLB may be optimized by improving working memory simultaneously with visuospatial processing skills, as opposed to targeting only visuospatial skills. Additionally, research measuring visuospatial skills and GLB should consider controlling for working memory. Furthermore, assessment practices of GLB may be advanced by identifying that the anatomical mechanisms of GLB may be disease specific.

## Ethics Statement

The study was approved by the SingHealth Centralized Institutional Review Board (CIRB) and conducted in accordance with Singhealth CIRB guidelines and the Declaration of Helsinki. Written informed consent was obtained from all subjects or their next of kin if they were mentally incapable of giving consent.

## Author Contributions

CY: study concept, study design, statistical analysis, interpretation of data, and preparation of manuscript; DL: study design, data processing, and preparation of manuscript, LL: data collection and preparation of manuscript; JZ: revision of manuscript for intellectual content; NK: study concept and revision of manuscript for intellectual content.

## Conflict of Interest Statement

The authors declare that the research was conducted in the absence of any commercial or financial relationships that could be construed as a potential conflict of interest.
