# Supplementary material for: Getting Lost Behavior in Patients with Mild Alzheimer’s Disease: A Cognitive and Anatomical Model
Source: Front Med (Lausanne). 2017 Nov 16;4:201. doi: 10.3389/fmed.2017.00201 (PMC5696332; doi:10.3389/fmed.2017.00201)
Supplement: Supplementary file 1 [file data_sheet_1.docx]

**Supplementary material**

**Methods**

*Primary outcome measure*, GLB was indexed using a semi-structured clinical interview with a psychologist blinded to diagnosis. Subjects were asked a) can you please describe how you travelled to the hospital today; b) do you still travel around on your own by walking, driving or taking public transport? if yes, what are the places that you frequently visit?; c) can you describe how you would map your route from your house to the places that you mentioned; d) do you realize that you are making wrong turns on paths that you have been travelling on for a long time, and e) have there been any instances whereby you got lost and if yes, how many times has it happened over the last 6 months? All subjects’ answers had to be corroborated by a caregiver, or family member in the case of controls, to contribute towards the GLB diagnosis. For caregivers/family members, the interview asked f) does the subject still drive or travel on their own?; g) have you observed that the subject experiences any way-finding difficulties?; h) do you realize that the subject has been making wrong turns on paths that they have been travelling on for a long time?, and i) have there been any instances whereby the subject got lost and if yes, how many times has it happened over the last 6 months? Subjects were diagnosed as experiencing GLB if they answered yes to questions a, b or c, with clear indications that they were not able to orientate themselves in familiar environments; or if they answered yes to questions d or e, indicating that there have been instances of getting lost; or if the caregiver/ family member responded yes to either f, g, h or i, indicating that there were instances whereby the subject had gotten lost.

**Image acquisition and processing**

3D volumetric scans were obtained using a T1-weighted magnetization-prepared rapid gradient-echo (MPRAGE) sequence (repetition time (TR) 2300ms, echo time (TE) 2.98ms, matrix=192x256×256, voxel=1.0mm isotropic) as per the AD Neuroimaging Initiative (ADNI) protocols (<http://adni.loni.usc.edu/>).

**Statistical analysis**

*Data preparation:* 10% of participants had missing GLB data and was thus imputed based on the recommendations by the American Psychological Association Task Force on Statistical Inference (Wilkinson, 1999) given that dropping non-random missing data has been found to bias the sample (Baraldi & Enders, 2010; Langkamp, Lehman, & Lemeshow, 2010). Missing data was imputed using multiple imputations with the five chained equations procedure to perform logistic regression analysis with the original weights. Composite scores for working memory, executive functions and visuospatial processing were calculated by averaging the z scores of each test.
